# Octopaminergic modulation of temporal frequency tuning of a fly visual motion-sensitive neuron depends on adaptation level

**DOI:** 10.3389/fnint.2015.00036

**Published:** 2015-05-26

**Authors:** Janina Lüders, Rafael Kurtz

**Affiliations:** Department of Neurobiology, Faculty of Biology, Bielefeld UniversityBielefeld, Germany

**Keywords:** adaptation, invertebrate, neuromodulation, octopamine, state dependence, velocity tuning, visual motion

## Abstract

Several recent studies in invertebrates as well as vertebrates have demonstrated that neuronal response characteristics of sensory neurons can be profoundly affected by an animal’s locomotor activity. The functional consequences of such state-dependent modulation have been a matter of intense debate. In flies, a particularly interesting finding was that tethered walking or flying causes not only general response enhancement of visual motion-sensitive neurons, but also broadens their temporal frequency tuning towards higher values. However, in other studies such state-dependent alterations of neuronal tuning functions were not found. We hypothesize that these discrepancies were due to different adaptation levels of the motion-sensitive neurons, resulting from the use of different stimulation protocols. This is plausible, because the strength of adaptation during ongoing stimulation was shown to be affected by chlordimeform (CDM), an agonist of the insect neuromodulator octopamine, which mediates state-dependent modulation. Our results show that CDM causes broadening of the temporal frequency tuning of the blowfly’s visual motion-sensitive H1 neuron only in the adapted state, but not prior to the presentation of adapting motion. Thus, our study indicates that seemingly conflicting results on the locomotor state-dependence of neuronal tuning functions are consistent when considering the neurons’ adaptation level. Moreover, it demonstrates that stimulation history has to be considered when the significance of state-dependent modulation of sensory processing is interpreted.

## Introduction

During transitions between different states of locomotor activity an animal’s sensory systems are confronted with prominent changes in the intensity as well as in the temporal profile of their input. Such behavioral-state-dependent changes of sensory input are particularly prominent for the processing of visual motion signals. During immobility the only source of visual motion input is motion resulting from movements of objects or other animals in the environment. In contrast, optic flow induced by self-motion can be the major source of visual motion input during walking or flight. State-dependent modulation of neuronal properties has been demonstrated in the visual system of different animal species (Rind et al., 2008; Chiappe et al., 2010; Maimon et al., 2010; Niell and Stryker, 2010; Jung et al., 2011; Keller et al., 2012; Ayaz et al., 2013; Longden et al., 2014). This modulation was argued to adjust the limited working range and the filtering properties of neurons to the stimulus properties during different locomotor states (Chiappe et al., 2010; Jung et al., 2011), and to help economize limited energy resources (Longden et al., 2014).

In the fly brain optic flow is processed by the tangential cells of the lobula plate (LPTCs). In Drosophila as well as in blowflies modulation by the locomotor state (resting *vs*. tethered flight or walking) has been demonstrated for several LPTCs, which can be individually identified (Chiappe et al., 2010; Maimon et al., 2010; Rosner et al., 2010; Jung et al., 2011; Suver et al., 2012). Moreover, similar alterations of response properties of LPTCs as induced by locomotor activity were evoked by administration of the insect neuromodulator octopamine or its agonist chlordimeform (CDM; Longden and Krapp, 2009, 2010; Chiappe et al., 2010; Jung et al., 2011; Suver et al., 2012). One of these alterations is particularly interesting, because it directly affects the computation of motion velocity: the increase in neuronal response gain by locomotor activity or CDM was much stronger for high than for low temporal frequencies of pattern motion (Chiappe et al., 2010; Jung et al., 2011; Longden et al., 2014). It was suggested that such an alteration of the neuron’s tuning function results from state-dependent differences in the temporal filtering properties within the basic units of motion computation, the so-called elementary motion detectors (Jung et al., 2011). From a functional point of view a particularly strong response boost for high temporal frequencies would make sense, because higher image velocities are present during walking or flight compared to resting. A similar effect of locomotor activity on temporal tuning functions was reported for neurons in the mouse visual cortex (Andermann et al., 2011).

However, a stronger octopamine-induced response boost for high than for low temporal frequencies of grating motion was not found in all studies (see e.g., Longden and Krapp, 2009; Suver et al., 2012). The effect appears more likely to be observed when steady-state neuronal responses were evaluated than when response transients briefly after motion onset were in the focus of data analysis. On the other hand, adaptation of LPTCs, which is expressed as a gradual decline of the neuronal response over time during sustained stimulation, was recently shown to depend on octopaminergic modulation (de Haan et al., 2012; Rien et al., 2012). This finding might explain why different conclusions about whether or not temporal frequency tuning is altered by octopamine were drawn. Usually, the adaptation-induced decline of the responses is particularly pronounced when the adaptor has a high temporal frequency (Harris et al., 2000; Kurtz et al., 2000; Reisenman et al., 2003). However, adaptation was shown to be less strong in the presence of CDM (see Rien et al., 2012; but see de Haan et al., 2012 for contrary results in hover flies). Taking together the effects of adaptation and CDM, one would expect to find the most prominent effect of CDM after strongly adapting stimulation, i.e., stimulation with a high temporal frequency. Since CDM counteracts the effect of adaptation, the strongest response boost by CDM would result for the range of high temporal frequencies. As a consequence, the temporal frequency tuning would be altered, much as observed in several studies (Chiappe et al., 2010; Jung et al., 2011; Longden et al., 2014). Noteworthy, no change of temporal frequency tuning could be observed when evaluating responses in the non-adapted state, i.e., response transients at motion onset (as in Longden and Krapp, 2010; Suver et al., 2012).

Thus, the state dependence of adaptation might be a major reason for the large differences in the magnitude of neuromodulation of fly LPTCs as well as in its qualitative effect on temporal frequency tuning curves observed in different studies. Across these studies, heterogeneous stimulation protocols and evaluation time windows were used, which likely resulted into different adaptation levels. In the present study we analyzed systematically how octopaminergic modulation interacts with adaptation by using a reference-adapt-test stimulus protocol. Specifically, we addressed the question whether CDM-induced modulation of the temporal frequency tuning of the H1 neuron, one of the blowfly’s LPTCs, depends on adaptation level.

## Material and Methods

### Animal Preparation and Electrophysiology

Female blowflies (*Calliphora vicina*), taken from our laboratory stock, were briefly sedated with CO_2_ to fix them with bee’s wax at the dorsal thorax to a small glass plate. The legs were removed and the wounds were covered with wax. The head was pitched downwards and the proboscis was extended, both being fixed in place with wax. The right lobula plate was exposed by opening the head capsule and by removing fat tissue and some main tracheae, if necessary. Ringer’s solution (composition in mM: NaCl 128.3, KCl 5.4, glucose 13.9, NaHCO_3_ 4.8, KH_2_PO_4_ 3.4, CaCl_2_ 1.9, pH 7.0; all chemicals from Merck, Darmstadt, Germany) was used to prevent desiccation of the brain and to fill a glass pipette that served as indifferent electrode. All experiments were performed at temperatures ranging from 20 to 30°C. The H1 cell receiving visual input from the left visual field was recorded in its output arborization in the right lobula plate. H1 was identified by its large receptive field in which it responds with high spike rates to back-to-front motion (Eckert, 1980; Krapp et al., 2001). Spikes were registered by extracellular electrodes pulled from borosilicate glass (GC150TF-10, Clark Electromedical, Edenbridge, UK) with a Brown–Flaming electrode puller (P97, Sutter Instruments, San Rafael, CA, USA) and filled with 1M KCl. Electrical signals were amplified and filtered by custom-built equipment. The signals were sampled at 25 kHz by an I/O card (DT 3001; Data Translation, Marlboro, MA, USA) and stored for offline analysis via routines written in Matlab R2013a (The MathWorks Inc., Natick, MA, USA).

### Visual Stimulation

**Figure 1:**
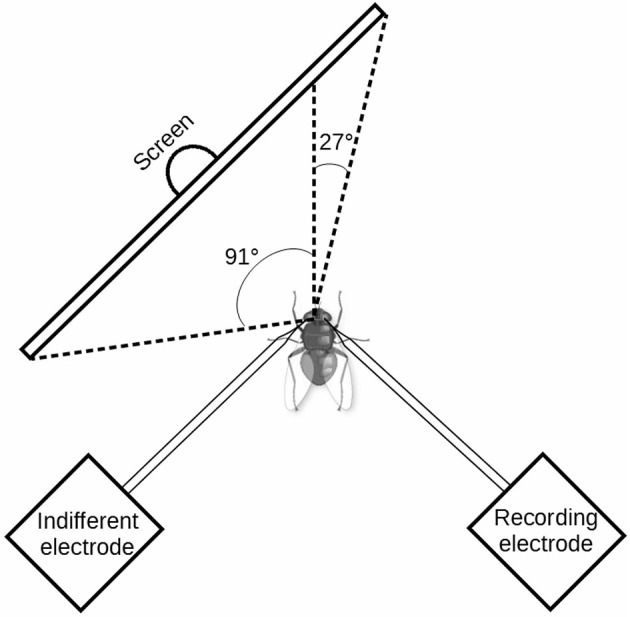
**Scheme of the experimental setup seen from above**. Drifting sinewave grating were presented to the fly on a 120 Hz thin-film transistor (TFT) monitor. The H1 neuron receives its major input from the visual field contralateral to the recording side. Along the elevation axis, the monitor covered 37° in the dorsal as well as in the ventral field of view. Note that, in order to provide access to the recording region the axis of fly’s thorax and abdomen was bent down relative to the head axis. For illustrative reasons this detail is not shown in the scheme.

Drifting sine-wave gratings were displayed on a thin-film transistor (TFT) monitor (Samsung Syncmaster 2233, Samsung Electronics Co., Yongin, Korea) at a frame rate of 120 Hz. Stimuli were designed with self-written programs using OpenGL/Vision Egg (Straw, 2008). Pattern motion was always perpendicular to the stripe orientation of the grating. The visual angles covered by the stimulus patterns along the azimuth were approximately 91° and 27° in the contralateral and ipsilateral fields of view of the fly, respectively (see Figure 1). Along the elevation axis, the angle was 37° in the dorsal as well as in the ventral field of view. The spatial wavelength of the sine-wave grating was 10°. The mean luminance of the grating was 32 cd/m^2^ and the Michelson contrast was 0.97. We used a reference-adapt-test protocol with various temporal frequencies to determine how the temporal frequency tuning of the H1 neuron is affected by adaptation and by octopaminergic neuromodulation (see methods section “Pharmacology” below). The stimulus sequence consisted of presentation of a gray screen with mean luminance for 1.6 s, reference motion for 1 s, a gray screen with mean luminance for 0.5 s, adaptation motion for 6 s, a gray screen with mean luminance for 0.5 s, and test motion for 1 s. The temporal frequency of reference and test stimuli was always the same, selected from the following set of temporal frequencies: 0.25 Hz, 0.5 Hz, 1 Hz, 2 Hz, 4 Hz, 8 Hz, 16 Hz, 32 Hz. For the adaptation stimuli three conditions were tested: 0.5 Hz, 8 Hz, and a condition in which the temporal frequency during adaptation was equal to that of the reference/test stimuli. All stimulus parameters were selected in pseudo-random order. Consecutive stimulus presentations were separated by an interval of at least 5 s, in which a gray screen with mean luminance was shown. It should be noted that during presentation of the highest temporal frequency (32 Hz) the temporal profile of the local brightness modulations showed deviations from an exact sine wave. When measured with a photodiode, the smoothness of the signal was seen to be corrupted by step-like changes in brightness, as expected given the monitor’s refresh rate of 120 Hz. Nevertheless, we decided to include the data for 32 Hz, because these imprecisions in stimulus presentation affect the neuronal responses under all experimental conditions equally. Thus, they are likely to result into increased variability for the 32-Hz stimulus, but not into systematic differences between the tested states of adaptation and neuromodulation.

### Pharmacology

To mimic the effects of octopamine, we applied the tissue-permeable octopamine receptor agonist CDM-HCl (CDM; Sigma-Aldrich, Dorset, UK; Evans and Gee, 1980; Hollingworth and Murdock, 1980). CDM was dissolved in water (aqua bidest.) as a 0.26 mM stock solution and stored at −18°C. Before each experiment, the CDM stock solution was diluted in fly Ringer solution (for compostion see methods section “Animal preparation and electrophysiology” a*bove)* to obtain a final concentration of 2.6 μM. A drop of 10 μL CDM solution was applied to the fly’s brain (see Longden and Krapp, 2009; Rien et al., 2012 for discussions on why this concentration and amount was chosen). We waited at least 10 min for CDM to become effective before resuming our recordings.

### Data Analysis

The data was analyzed with routines written in Matlab. For all results presented in this study the number of neurons recorded ranged from 7 to 13. In each fly, the number of visual stimulus presentations for each stimulus condition usually was 4. In some cases, it was reduced to 3, if response trials had to be removed due to artifacts or low signal-to-noise ratio. The time points of action potentials were determined by a threshold, which was adjusted for each fly. Data were collected only from neurons that displayed sufficiently large extracellular spike amplitudes relative to the amplitude distribution of background noise, resulting in signal-to-noise ratios of at least 2.5:1. Spike frequencies were calculated based on the inverse of the duration of inter-spike intervals and displayed as peri-stimulus time-histograms (PSTHs) with a bin width of 20 ms. Response magnitudes under different stimulus conditions were quantified in the following way: first, baseline spike rate was calculated in a 1-s period prior to the reference stimulus and subtracted; second, baseline-subtracted responses in the unadapted and adapted state were averaged in 200-ms time windows starting 50 ms after onset of the reference and test stimulus, respectively.

Data statistics was based on non-parametric testing, because several tests for normality (Shapiro-Wilk-Test, Anderson-Darling-Test, Lilliefors-Test) uniformly indicated that the assumption of normal distribution of the data is violated in some of the conditions. The Scheirer-Ray-Hare-Test (SRH test; Scheirer et al., 1976), a non-parametric alternative to ANOVA with repeated measures, was used to examine which of the factors, visual stimulus conditions, adaptation state (before vs. after adaptation) and pharmacological treatment (before vs. after administration of CDM), and which of their pair-wise interactions, have a significant effect on the measured spike rates. We applied three-way SRH tests, in which spike rate was considered the dependent variable, whereas neuron identity and two of the factors were considered independent variables. This test scheme resulted into the following three combinations of independent variables: stimulus/adaptation/neuron identity; stimulus/CDM/neuron identity; adaptation/CDM/neuron identity. A result was considered significant given a *p* value < 5%.

Statistical significance of differences between adaptation state and pharmacological treatment were tested *post hoc* for each of the visual stimulus conditions by the Wilcoxon two-sided signed rank test for paired data, considering a significance level of *p* < 5%. To account for the problem of multiple comparisons the Benjamini-Hochberg procedure was applied (Benjamini and Hochberg, 1995). Following this procedure the null hypothesis is rejected only for test results that follow the criterion *p* < (i/m)*Q, with: *p* = *p* value of Wilcoxon test, i = rank of *p* value (*i* = 1 for smallest *p* value, *i* = 2 for second smallest *p* value, etc.), m = total number of tests, Q = false discovery rate. The Q value was set to 5%.

## Results

To examine whether state-dependent alteration of neuronal response functions depends on adaptation level we determined the temporal frequency tuning of the blowfly’s motion-sensitive H1 neuron before and after presentation of adapting motion. We used a reference-adapt-test stimulus protocol, in which the responses to the reference stimulus can be taken to approximate the unadapted response, whereas the responses to the test stimuli represent responses in an adapted state. Note however that time windows for evaluation of neural responses cannot be ultimately short. Thus, even for the “unadapted” response some adaptation is likely to be caused by the reference stimulus itself, because adaptation of LPTCs was shown to operate on a remarkably fast timescale (Nordström et al., 2011). To mimic different locomotor states we used systemic application of CDM, an agonist of octopamine, which was shown to be released during flight and to induce state-dependent changes of responsivity of fly LPTCs (Suver et al., 2012).

**Figure 2:**
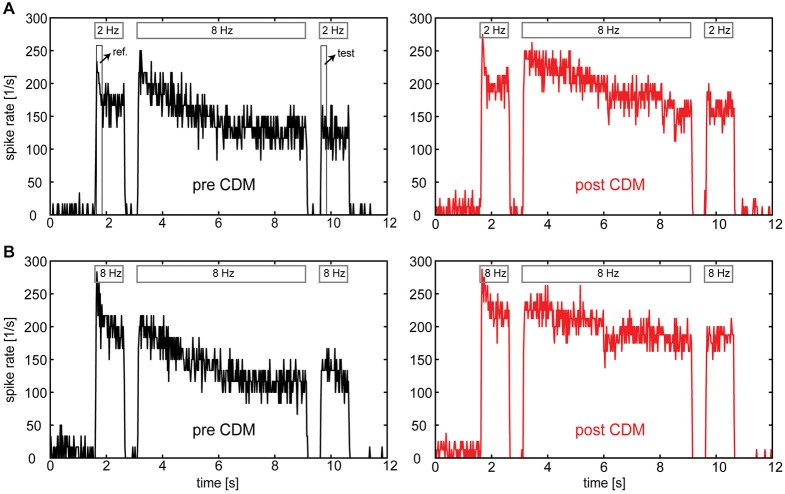
**Responses of the H1-neuron to motion in the preferred direction before and after administration of CDM**. PSTHs were calculated from spike traces of a single neuron in a reference-adapt-test protocol. **(A)** Temporal frequencies of 2 Hz and 8 Hz were used for reference/test motion and adapting motion, respectively. **(B)** A temporal frequency of 8 Hz was used for the entire stimulus protocol. Bars on top of each response trace indicate the duration of reference, adapt and test motion. Boxes in the upper left diagram indicate the time windows for the calculation of unadapted (“ref.”) and adapted (“test”) responses. Note that the discretization in spike rate is not a genuine neuronal property, but due to limited numbers of sweeps (*n* = 3–4) per condition together with 20-ms binning of the data.

Figure 2 shows examples of PSTHs calculated from the responses of an H1-neuron before and after administration of CDM. In all cases the temporal frequency of adapting motion was 8 Hz. In Figure 2A reference and test stimuli had a temporal frequency of 2 Hz, whereas in Figure 2B the temporal frequency was 8 Hz. Two aspects of the data show that CDM counteracts the effects of adaptation. First, the decline of the response level during the adaptation period is less pronounced after CDM administration compared to before. Second, the difference between the responses to the reference and to the test stimulus is reduced after CDM administration. These results match previous studies completely, which reported similar effects of CDM on the response time course (Longden and Krapp, 2010; Jung et al., 2011) and on the strength of adaptation (Rien et al., 2012). However, a third observation, not yet reported in previous studies, can be made when inspecting the PSTHs of Figure 2 in detail: for the 8 Hz test stimulus (i.e., after adaptation) the effect of CDM on the response appears to be slightly more pronounced than for the 2 Hz test stimulus. In contrast, for the responses to the 2 Hz and 8 Hz reference stimuli (i.e., before adaptation) the effects of CDM are similar. In the following, it is analyzed systematically whether adaptation level differentially affects the impact of CDM on the responses to different temporal frequencies.

By using a range of temporal frequencies between 0.25 Hz and 32 Hz in the reference and test period we determined temporal-frequency tuning-curves in the unadapted and in the adapted state. These tuning functions were obtained before and after administration of CDM. Moreover, we tested three different types of adapting motion: two data sets with either low or high temporal frequency during adaptation, 0.5 Hz and 8 Hz, respectively, and a third data set, in which the temporal frequency of adapting motion was the same as the one used in the reference and test periods. Compared to the first two conditions, the third condition is less well suited for systematical tests of how adaptation and CDM affect temporal frequency tuning, because the adaptation state of the neuron differs for the different test stimuli. Nevertheless, we decided to include this condition, because it closely matches the conditions of previous studies, in which single motion stimuli (i.e., not divided into reference, adapt and test stimuli) with different temporal frequencies were presented. When choosing an analysis time window several seconds after motion onset, as has been done in some of these studies, the adaptation state inevitably differs across conditions, because different temporal frequencies differ in their effectiveness to induce adaptation.

**Figure 3:**
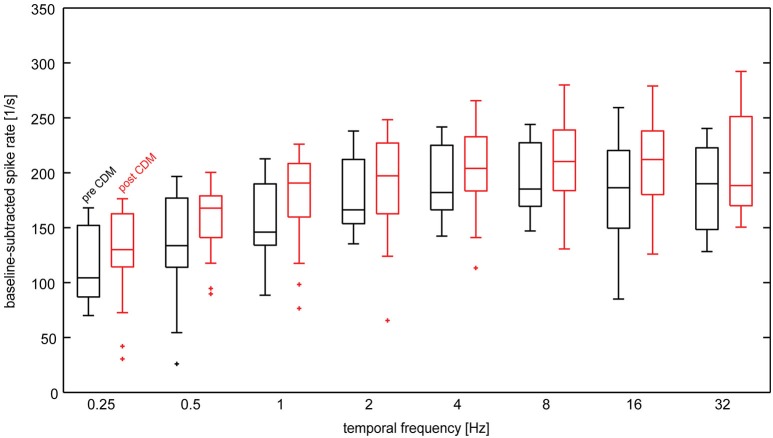
**Temporal frequency tuning of the H1-neuron in the unadapted state before and after administration of CDM**. Baseline-subtracted spike rates were calculated from the responses to the reference stimulus. Data from the different adaptation protocols used in the study was lumped together, because the reference stimulus precedes the adaptation phases. Data are depicted as box-whisker plots, in which the central line indicates the median, horizontal box boundaries indicate upper and lower quartiles and the whiskers show the extent of the rest of the data. Maximal whisker length is 1.5 times the inter-quartile range. Data values beyond the end of the whiskers are classified as outliers and are displayed ascrosses. Sample size 7–13 neurons.

Figure 3 shows the temporal frequency tuning of H1 neurons before and after administration of CDM in the unadapted state, that means, in a 200-ms time windows starting 50 ms after onset of the reference stimulus. The tuning functions before as well as after administration of CDM are bell-shaped, with a peak in the range 4–16 Hz. CDM leads to an elevation of the median response levels by about 30–50 spikes/s. This response enhancement by CDM is largely independent of the temporal frequency.

**Figure 4:**
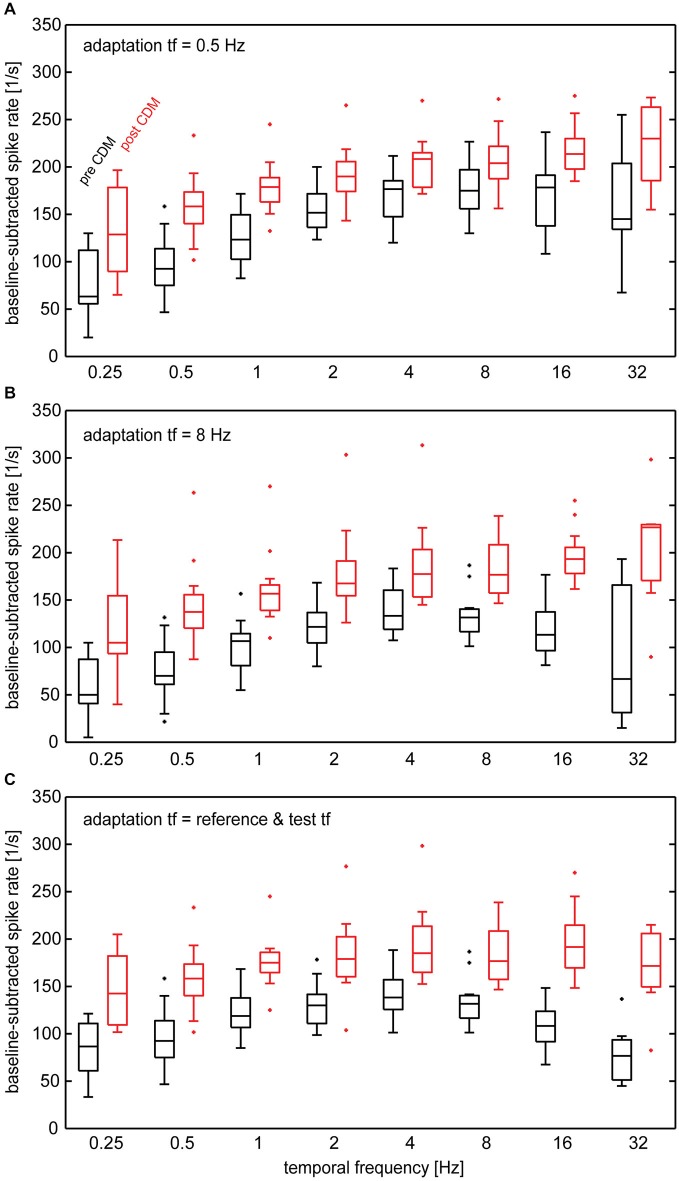
**Temporal frequency tuning of the H1-neuron in different adapted states before and after administration of CDM**. Baseline-subtracted spike rates were calculated from the responses to test stimuli following adapting motion. **(A)** Temporal frequency of adapting motion was 0.5 Hz. **(B)** Temporal frequency of adapting motion was 8 Hz. **(C)** Temporal frequency of adapting motion was the same as that in the reference/test phases. See legend of Figure 2 for an explanation of box-whisker plots. Sample size 7–13 neurons.

Next, we determined the effect of CDM on the temporal frequency tuning following adaptation by examining the responses to the test stimuli. Compared to the unadapted state, the increase in response gain by CDM in the adapted state depends on temporal frequency (Figure 4). Although responses are enhanced by CDM for all temporal frequencies, the gain enhancement is strongest for high temporal frequencies (8–32 Hz). This results in prominent changes of the shape of the temporal frequency tuning. Before administration of CDM, the tuning curves obtained after adaptation with 0.5 Hz (Figure 4A) and 8 Hz (Figure 4B) peak in the range of 4–16 Hz and 2–8 Hz, respectively. After administration of CDM, for both adaptation conditions the tuning-curve peaks lie close to the highest temporal frequency tested, 32 Hz, or even outside this range. A similar pattern emerges when the temporal frequency of adapting motion equals that of the reference/test stimuli (Figure 4C). In this case CDM application leads to a shift of the tuning curve peak from about 2–8 Hz to about 4–16 Hz.

**Figure 5:**
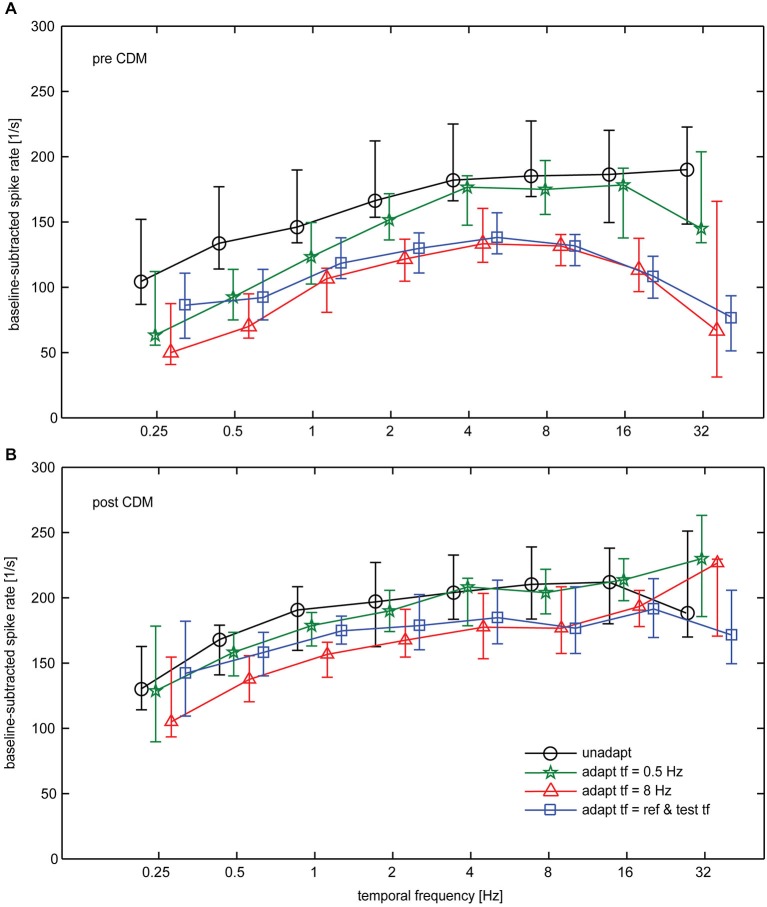
**Impact of CDM on temporal frequency tuning of the H1-neuron summarized for all conditions. (A)** Temporal frequency tuning before administration of CDM. **(B)** Temporal frequency tuning after administration of CDM. Symbols and error bars show the median and the inter-quartile range of the data. Sample size 7–13 neurons.

In Figure 5 the tuning curves for all the tested conditions are compiled for the data obtained before (Figure 5A) and after (Figure 5B) CDM application. Statistical testing showed that, in general, CDM has a significant effect on the spike rates (SRH test, *p* < 0.0001). Further factors that significantly influence neuronal activity are adaptation state, stimulus condition and neuron identity (SRH tests, all *p* < 0.0001). Out of the possible pair-wise interactions between these factors only that between CDM and neuron identity reached statistical significance (*p* = 0.0022). This significant interaction suggests that CDM might have been unequally effective across our preparations. The results of *post hoc* Wilcoxon signed rank tests for the different stimulus conditions are shown in Table 1.

**Table 1:** **Statistical test results**.

Adapt TF [Hz]	Test TF [Hz]	Adapted *vs*. unadapted (before CDM)	Adapted *vs*. unadapted (after CDM)	Before CDM *vs*. after CDM (unadapted)	Before CDM *vs*. after CDM (adapted)
0.5 Hz Adaptation					
0.5	0.25	***0.01563***	0.57813	0.29688	***0.01563***
0.5	0.5	***0.00024***	0.89258	0.08032	***0.00024***
0.5	1	***0.00049***	0.90844	***0.01343***	***0.00024***
0.5	2	***0.00122***	0.89258	0.19092	***0.00170***
0.5	4	***0.01049***	0.94604	0.14648	***0.00024***
0.5	8	***0.02099***	0.73535	0.19092	***0.00073***
0.5	16	**0.02563**	0.21631	0.06812	***0.00024***
0.5	32	0.21875	0.29688	0.37500	**0.03125**
8 Hz Adaptation
8	0.25	***0.01563***	0.68750	0.56250	**0.03125**
8	0.5	***0.00049***	0.21631	0.09424	***0.00024***
8	1	***0.00024***	0.05737	***0.02051***	***0.00024***
8	2	***0.00024***	0.19092	0.07813	***0.00024***
8	4	***0.00024***	0.12719	0.33959	***0.00049***
8	8	***0.00024***	0.08032	0.16772	***0.00024***
8	16	***0.00073***	0.19092	***0.00342***	***0.00024***
8	32	***0.01563***	0.93750	0.29688	***0.01563***
Adaptation tf = test tf
0.25	0.25	***0.01563***	0.57813	0.23438	***0.01563***
0.5	0.5	***0.00024***	0.89257	0.08032	***0.00024***
1	1	***0.00073***	0.94604	0.08032	***0.00024***
2	2	***0.00024***	0.21631	0.16772	***0.00049***
4	4	***0.00024***	0.12719	***0.02148***	***0.00024***
8	8	***0.00024***	0.08032	0.16772	***0.00024***
16	16	***0.00049***	0.33959	0.14648	***0.00024***
32	32	***0.01563***	0.07813	0.10938	***0.01563***

*SRH-tests indicated that spike rates are significantly affected by the factors stimulus condition, adaptation state, CDM treatment and neuron identity (p < 0.0001 for all factors). The table shows error probabilities determined post hoc by Wilcoxon two-sided signed rank tests for paired data. Results considered significant beneath a significance level of p < 5% are highlighted by bold font. Results for which statistical significance is preserved after application of the Benjamini-Hochberg correction for multiple comparisons with a corresponding false discovery rate of 5% are highlighted by italic bold font*.

It becomes evident from the curves shown in Figure 5 that, in general, CDM induces a broadening towards high temporal frequencies. However, whereas this effect is present for any of the adapted states, it is absent in the unadapted state. If at all, CDM affects neuronal tuning in the unadapted state in an opposite way to what is found for the adapted state, that means by shifting temporal frequency tuning towards lower values. Figure 5A also reveals that before CDM administration temporal frequency tuning is altered by adaptation. In particular, after adaptation with 8 Hz as well as after adaptation with a temporal frequency that is equal to that of the test stimuli, the adaptation-induced attenuation of the responses is stronger for high than for low and medium temporal frequencies. This effect of adaptation is reduced after application of CDM, leading to largely similar temporal frequency tunings in the unadapted and the differently adapted states (Figure 5B). The higher similarity of temporal tuning curves after CDM administration compared to before is also reflected in the statistics of our data (see Table): before CDM, significant differences between responses in the unadapted and the adapted state exist for almost all the conditions (Table 1, column 3). After CDM applications, none of the differences between unadapted and adapted state are statistically significant any more (Table 1, column 4).

**Figure 6:**
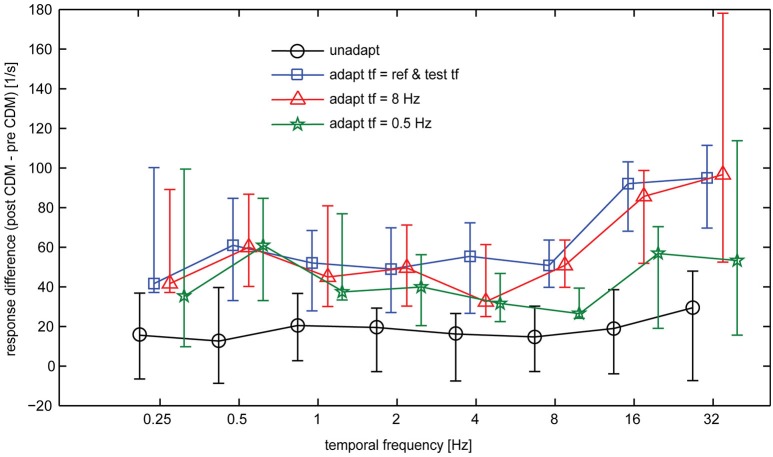
**CDM-induced differences in the baseline-subtracted spike rates of the H1-neuron**. Symbols and error bars show the median and the inter-quartile range of the data. Sample size 7–13 neurons.

Overall, our data show that CDM induces a particularly strong gain increase for the responses to high temporal frequencies in the adapted state, whereas in the unadapted state the effect of CDM is more evenly distributed across the entire range of temporal frequencies. Figure 6 summarizes the effect of CDM in the unadapted state and after different adaptation protocols by plotting the differences in spike rates induced by CDM across the range of temporal frequencies.

In general, the CDM-induced enhancement of the responses is stronger after adaptation than before adaptation. This is also reflected in the statistics of the data, which indicates significant differences before and after CDM application for all stimulus conditions in the different adapted states, but only for some of the conditions before adaptation (Table 1, columns 5 and 6). Moreover, in the adapted state, the data indicates a dependency of CDM-induced differences on temporal frequency. Whereas this difference is similar for all temporal frequencies in the unadapted state, after adaptation slightly larger differences are obtained for 16 and 32 Hz than for medium and low temporal frequencies. This dependency on temporal frequency is most pronounced for adaptation with 8 Hz and when the temporal frequency of the adapting stimulus and the test stimuli were equal, but only weak for adaptation with 0.5 Hz.

## Discussion

In this study we examined the stimulus conditions leading to general state-dependent modulation of neuronal response gain on the one hand and to specific alterations of neuronal tuning functions on the other. This difference is highly relevant in a functional context. Although general modulation of response gain might be valuable as well, for example for economizing energy expenditure for neural signaling, state-dependent modulation of neuronal tuning is particularly fascinating. Only the latter would enable animals to specifically adjust their neuronal computations to the particular requirements in a certain locomotor state. For fly LPTCs, locomotor activity was found to broaden the temporal frequency tuning towards high values (Chiappe et al., 2010; Jung et al., 2011; Longden et al., 2014; Schnell et al., 2014). This state-dependent alteration of temporal frequency tuning was argued to be functionally beneficial, because it would allow accommodating for the higher prevalence of fast motion velocities during locomotion compared to rest (Longden and Krapp, 2011). However, in fly LPTCs other studies found state-dependent modulation of neuronal gain to be uniform across different temporal frequencies (Longden and Krapp, 2010; Suver et al., 2012). Although differences in fly species and cell types might in principle account for this discrepancy, it is more likely that different protocols for visual stimulation and data evaluation have led to different conclusions. It is noticeable, that in all studies, in which a state-dependent change in temporal frequency tuning was observed, the neural responses were evaluated in time windows starting at least one second after motion onset. For example, Jung and colleagues (Jung et al., 2011) took average responses in a 1-s time window starting two seconds after motion onset. Chiappe and colleagues (Chiappe et al., 2010) used calcium imaging during pattern motion and quantified neuronal activity by the peak of the calcium response, which occurred several seconds after motion onset, because calcium responses are slower than the electrical response. Interestingly, a study reporting tuning shifts in mouse visual cortex (Andermann et al., 2011) also used calcium imaging. In contrast, when in another study on the same brain area spiking activity during brief motion periods was evaluated an overall gain increase by motor activity but no change in neuronal tuning was observed (Niell and Stryker, 2010).

A major difference when using a late evaluation time window compared to one immediately after motion onset is the different adaptation level of the neuron. The octopamine agonist CDM was shown to counteract the effect of adaptation in the neurons V1 and H1 of blowflies (Rien et al., 2012). Therefore, we expected to find stronger effects of CDM in the adapted state than in the unadapted state. The results of the present study are consistent with this expectation. Moreover, a strong impact of CDM on neuronal responses would be expected for stimuli that cause strong adaptation. This effect can also be seen in the data: adaptation with 8 Hz causes stronger response attenuation than adaptation with 0.5 Hz, and, accordingly, the effect of CDM also appears to be more pronounced after adaptation with 8 Hz (see Figure 5). Most earlier studies in which state-dependent alteration of temporal frequency tuning was investigated did not use a reference-adapt-test protocol, but a single motion period with uniform temporal frequency (Chiappe et al., 2010; Jung et al., 2011; Schnell et al., 2014). With such a protocol it is inevitable that the adaptation level is different for different temporal frequencies. If state-dependent modulation interferes with adaptation, apparent differences in the strength of state-dependent modulation across the temporal frequency range might thus directly result from the different adaptation level induced by different temporal frequencies of pattern motion. However, in the present study, CDM was also able to broaden the temporal frequency tuning towards high values when a reference-adapt-test protocol was used, in which the temporal frequency of the adaptating stimulus was exactly the same for all test stimuli (see Figures 5A,B). Thus, alteration of temporal frequency tuning by CDM appears to be a genuine effect, rather than an “artefact” of making comparisons across different adaptation levels. A possible reason could be that adaptation itself alters temporal frequency tuning by differentially affecting the response to different temporal frequencies (see Figure 5A). If this change of temporal frequency tuning by adaptation is counteracted by CDM, the effect of CDM itself is also temporal-frequency dependent.

Our present results are consistent with a study, in which walking activity was found to alter the tuning of the responses of the blowfly H2 neuron to test stimuli with various temporal frequencies after adapting with uniform temporal frequency (Longden et al., 2014). In contrast to our study, the adaptation stimulus consisted of motion in the neuron’s anti-preferred direction in Longden et al. (2014). At first sight, it might be surprising that adaptation in the neuron’s preferred and anti-preferred directions produce similar effects. However, this finding is consistent with earlier observations that the component of adaptation that was shown to be affected by CDM (de Haan et al., 2012; Rien et al., 2012), is induced irrespective of the direction of motion (Harris et al., 2000). Although the cellular mechanisms underlying octopaminergic modulation of adaptation are not known yet, the lack of direction selectivity indicates that it originates from changes presynaptic to LPTCs.

Although the major focus of the present study was the state-dependence of neuronal tuning functions, our results also reveal a functionally relevant aspect of adaptation, which was to our knowledge so far not described in previous studies. Temporal frequency tuning of the H1-neuron appears to be modified by adaptation. This is most evident after adaptation with 8 Hz, which results into a decrease of neuronal responsivity that is most pronounced for high temporal frequency (Figure 5A). Thus, the peak of the temporal frequency tuning is shifted towards lower values by adaptation with 8 Hz. In contrast, with 0.5 Hz adaptation the strongest response decrease is present for low temporal frequencies. Similar indications of “stimulus-specific-adaptation” have been described to affect velocity tuning of neurons in the primary visual cortex of cats (Hietanen et al., 2007). Interestingly, in the present study the stimulus-specific effects of adaptation appear to be counteracted by CDM (Figure 5B). Thus, our findings suggest that it might be necessary to re-evaluate adaptation effects reported in studies on inactive animals by performing recordings during locomotor activity or after pharmacological activation of arousal systems.

In summary, the present study shows that temporal frequency tuning of motion-sensitive neurons in the fly brain is differentially affected by octopaminergic modulation, depending on the neuron’s adaptation level. CDM was found to induce a broadening of the tuning function towards higher temporal frequencies in the adapted state, but not in the unadapted state. In contrast, an overall boost in neuronal gain was present irrespective of adaptation level. Thus, our study allows us to reconcile the seemingly conflicting evidence of previous studies on whether or not temporal frequency tuning is altered during locomotor activity (Chiappe et al., 2010; Jung et al., 2011; Suver et al., 2012; Longden et al., 2014; Schnell et al., 2014) or during artificial activation of octopaminergic signaling (Longden and Krapp, 2010; Jung et al., 2011; Suver et al., 2012). The results of our study are relevant to clarify under which stimulation conditions and in which functional contexts state-dependent modulation of neuronal tuning functions becomes effective.

## Author Contributions

J.L. performed the experiments, analyzed the data and contributed to drafting the manuscript. R.K. designed the research, contributed to data analysis and wrote the manuscript.

## Conflict of Interest Statement

The authors declare that the research was conducted in the absence of any commercial or financial relationships that could be construed as a potential conflict of interest.
